# Optimal Timing of Initial Surveillance MRI Following Treatment of Brain Metastases With Stereotactic Radiation: A Comparison of Six Weeks Versus Three Months

**DOI:** 10.7759/cureus.102720

**Published:** 2026-01-31

**Authors:** Emily P Vonderhaar-Meister, Timothy A Lin, Anne Li, Catherine Siu, Chetan Bettegowda, Shih-Chun Lin, Jordina Rincon Torroella, Jon D Weingart, Kristin J Redmond, Lawrence R Kleinberg

**Affiliations:** 1 Department of Radiation Oncology and Molecular Radiation Sciences, Johns Hopkins University School of Medicine, Baltimore, USA; 2 Department of Radiation Oncology, University of Texas MD Anderson Cancer Center, Houston, USA; 3 Renaissance School of Medicine, Stony Brook University, Stony Brook, USA; 4 Department of Neurosurgery, Johns Hopkins University School of Medicine, Baltimore, USA; 5 Reza Khatib Brain Tumor Research Center, Johns Hopkins Medicine, Baltimore, USA

**Keywords:** brain metastases, brain radiosurgery, follow-up surveillance imaging, mri, stereotactic radiotherapy

## Abstract

Patients with brain metastases are increasingly being treated with stereotactic brain radiation, in one fraction termed stereotactic radiosurgery (SRS) or two-five fractions termed fractionated stereotactic radiotherapy (FSR), due to its sparing of neurocognitive dysfunction without compromising survival benefit. However, distant brain failure rates remain high, and the optimal interval for post-radiation MRI surveillance is not well established. This study evaluates the potential clinical benefit of early MRI surveillance compared with a typical three-month timeline. The primary objective of this study was to compare radiographic and clinical outcomes associated with early six-week versus 12-week post-treatment MRI surveillance following stereotactic radiation for brain metastases. In this retrospective study of patients with one or more brain metastases treated with SRS or FSR at a single tertiary center, initial post-treatment surveillance MRI and clinical follow-up were compared between patients who underwent follow-up at a six-week interval versus a 12-week interval after radiation. Of 309 patients reviewed, 232 (75.1%) completed surveillance imaging; 123 (53%) underwent later timeline 12-week MRI with a median time to MRI of 92 days, and 109 (47%) underwent early timeline six-week MRI with a median time to MRI of 43 days (p < 0.001). Among patients in the 12-week MRI group, 52 (42.3%) had radiographical findings on MRI concerning new and/or progressive disease compared to 36 (33.0%) in the early MRI group (p = 0.18). There was similarly no statistically significant difference in patients with new or worsening neurologic symptoms during clinical follow-up in the 12-week (28 (22.8%)) versus early (28 (25.7%)) groups (p = 0.46). Following MRI surveillance, completion and types of subsequent clinical interventions were documented; the frequency of patients undergoing additional interventions did not differ significantly between groups (49 (39.8%) vs 33 (30.3%), p = 0.17). While the overall distribution of intervention type did not statistically differ among patients in either cohort, a higher proportion of those who underwent intervention in the early MRI group went on to receive a short-interval MRI based on equivocal findings at the time of initial MR, while a smaller proportion went on to receive additional radiation therapy. These data support current practice guidelines recommending routine MRI surveillance following SRS or FSR at three-month intervals, as early MRI surveillance did not significantly impact outcomes in this cohort. However, additional studies to identify patients at increased risk for early, or “high velocity”, distant brain progression who may benefit from earlier surveillance are warranted.

## Introduction

Stereotactic brain radiation is implemented for an increasing number of patients with brain metastasis, with randomized data showing equivalent survival and better quality of life for patients compared to previously standard whole brain radiation therapy (WBRT) [1-3]. However, close MRI monitoring is required as there is a substantial risk of new lesions developing in untreated brain [2-4]. Little information is available to guide decisions regarding follow-up imaging schedules to optimize outcomes and preserve resources. Here, we focus on whether there is clinical value to obtaining an initial follow-up MRI at six weeks post-treatment rather than three months, recognizing that the pace of development of additional lesions is uncertain after brain metastases requiring treatment appear.

Current guidelines recommend routine neuroimaging every three months for patients with a history of brain metastases [5]. Survival for patients with metastatic cancer, including with central nervous system (CNS) involvement, has increased over the years with the advent of improved systemic therapies, including immunotherapy [6]. As survival improves in these patients, frequent MRI follow-up can impose a significant patient burden, including time toxicity, financial strain, and emotional stress awaiting results [7]. However, the potential benefit of early MRI surveillance in identifying newly developed lesions before they cause neurological morbidity provides the rationale for this investigation. Indeed, early detection is clinically meaningful, as patients with symptomatic presentation of brain metastases demonstrate higher rates of neurologic death, prolonged hospitalizations, and greater healthcare costs, compared to patients with asymptomatic brain metastases discovered through routine surveillance [8]. At present, the optimal timing of initial MRI surveillance following stereotactic brain radiation to detect new lesions while asymptomatic and treatable by additional radiotherapy is unknown.

The primary objective of this study was to investigate whether early six-week initial surveillance imaging confers an advantage when compared to a typical 12-week timeline following stereotactic radiation treatment to brain metastases. Secondary objectives included the comparison of subsequent clinical interventions between surveillance strategies. To address these aims, radiographic and clinical outcomes at the initial follow-up period were analyzed in patients who underwent definitive or postoperative stereotactic brain radiation.

Portions of this article were previously presented as a poster at the 2021 Society for NeuroOncology (SNO) Conference on Brain Metastases on August 3, 2021 (initial cohort of 100 patients) and as a poster at the 2025 ACRO Summit on March 13, 2025, and the 2025 SNO Conference on Brain Metastases on August 14, 2025 (full cohort).

## Materials and methods

Study design and patient population

An IRB-approved retrospective study with waiver of consent was conducted of 309 consecutive patients with brain metastases treated with 1-5 fractions of stereotactic radiation in the definitive or postoperative setting at a single tertiary academic institution between 2017 and 2020. Radiation delivered in one fraction is termed stereotactic radiosurgery (SRS), and that delivered in two to five fractions is termed fractionated stereotactic radiotherapy (FSR). Inclusion criteria were as follows: patients aged 18 years and older, having one or more brain metastases treated with SRS or FSR, and with completed post-treatment surveillance MRI imaging. Patients without post-treatment follow-up MRI were excluded. The timing of the initial surveillance MRI scan was scheduled for approximately six weeks or 12 weeks following completion of radiotherapy, based on the follow-up policy of different providers. Standard diagnostic MRI sequences were obtained. Patients were grouped into one of two cohorts based on the interval from the initiation of radiotherapy to the date of the first surveillance MRI: patients who underwent imaging before 56 days were categorized in the six-week group, while those who received imaging on or after 56 days were categorized in the 12-week group.

Data collection

Clinical and demographic data were extracted from the electronic medical record, including age, sex, race, performance status (measured by Karnofsky Performance Status (KPS) and/or Eastern Cooperative Oncology Group (ECOG) performance scales), primary tumor histology, number and location of brain metastases, treatment modality (SRS vs. FSR), history of prior brain radiation, status of extracranial disease burden and details of systemic therapy when applicable. The date and findings of the initial surveillance MRI were recorded. MRI reports and relevant clinical notes were reviewed to determine clinical status and radiographic outcomes, which were stratified as (i) stable/improved disease, (ii) concern for new brain metastases, (iii) concern for progression of existing metastases, or (iv) concern for new and progressive brain metastases. The primary outcome of this study was detection of new or progressive intracranial lesions on initial surveillance MRI. Secondary outcomes included presence of new or worsening neurological symptoms at time of initial clinical follow-up, and whether surveillance imaging findings triggered clinical interventions assessed within three months of the first surveillance scan including repeat short-interval MRI (approximately 1-2 months), additional intracranial stereotactic radiation or WBRT, surgical resection of new or progressive lesions, addition of systemic therapy, or enrollment in hospice.

Statistical analysis

Descriptive statistics were used to summarize baseline characteristics. Comparative analyses between the six-week and 12-week cohorts were performed using chi-squared or Fisher's exact tests for categorical variables and t-tests or Mann-Whitney U tests for continuous variables, depending on distribution and variance. A p-value of <0.05 was considered statistically significant. All analyses were conducted using R v4.5.0 (R Foundation for Statistical Computing, Vienna, Austria).

## Results

**Figure 1:**
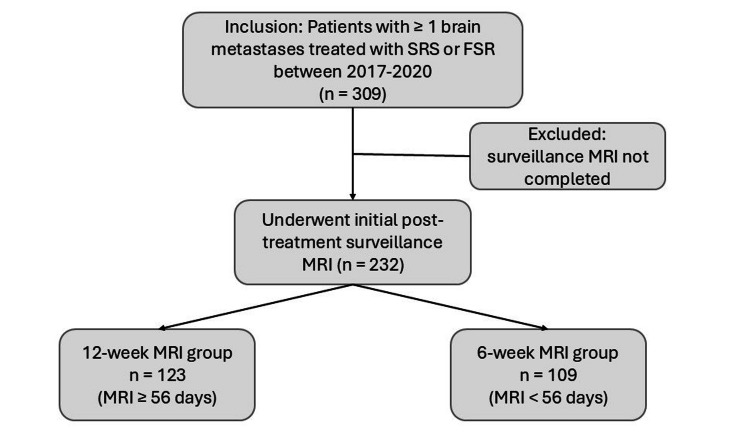
Study design.

Three hundred and nine patients with brain metastases treated with stereotactic radiation were reviewed. There were 232 (75.1%) eligible patients with 680 metastatic lesions included for analysis. Of these patients meeting inclusion criteria, 123 (53%) underwent 12-week MRI (median 92 days from treatment) and 109 (47%) underwent six-week MRI (median 43 days from treatment) (Figure 1, Table 1). Baseline demographics including age (p = 0.51), race and ethnicity (p = 0.40) and sex (p = 0.11) were comparable between groups. Performance status (PS) was categorized as good PS (ECOG 0-1 or KPS 70 or higher) or poor PS (ECOG 2+ or KPS 60 and lower) and was recorded for all but one patient; the cohorts similarly included a majority of patients with a good PS (108 (88%) and 95 (87%), p = 0.76).

**Table 1:** Patient demographics and clinical characteristics. IQR, interquartile range, NC, not controlled; NSCLC, non-small cell lung cancer; NR, not recorded; PCI, prophylactic cranial irradiation; SCLC, small cell lung cancer; SD, standard deviation; SRS, stereotactic radiosurgery; WBRT, whole brain radiation therapy.

Patient demographics and clinical characteristics	12-week MRI (n = 123)	6-week MRI (n = 109)	p-value
Median time to initial MRI scan in days (IQR)	92 (72-104)	43 (40-48)	< 0.001
Mean age in years (SD)	62.8 (11.3)	61.8 (11.9)	0.51
Race/Ethnicity (n (%))	-	-	0.4
White	88 (72)	86 (79)	-
Black	27 (22)	15 (14)	-
Asian	6 (5)	7 (6)	-
Hispanic	1 (1)	1 (1)	-
Other	1 (1)	0 (0)	-
Sex (n (%))	-	-	0.11
Female	78 (63)	57 (52)	-
Male	45 (37)	52 (48)	-
Performance status (n (%))	-	-	0.76
Good	108 (88)	95 (87)	-
Poor	15 (12)	13 (12)	-
NR	0 (0)	1 (1)	-
History of prior SRS (n (%))	14 (11)	20 (18)	0.14
History of prior WBRT or PCI (n (%))	8 (7)	12 (11)	0.25
				Extracranial disease control at treatment (n (%))	-	-	0.09
Controlled	44 (36)	44 (40)	-
NC Primary only	7 (6)	2 (2)	-
NC Oligometastatic	42 (34)	26 (24)	-
NC Widespread	30 (24)	37 (34)	-
Histology (n (%))	-	-	0.04
NSCLC	54 (44)	31 (28)	-
SCLC	6 (5)	3 (3)	-
Breast	20 (16)	23 (21)	-
Melanoma	14 (11)	25 (23)	-
Other	29 (24)	27 (25)	-

Patients with a history of prior brain radiation, either prior SRS (14 (11%) vs. 20 (18%); p = 0.14) or prior WBRT or prophylactic cranial irradiation (PCI) (8 (7%) vs. 12 (11%); p = 0.25) were included in this study, and there was not a significant difference between the cohorts (Table 1). Patterns of extracranial disease control at the time of treatment showed no statistically significant differences between groups (p = 0.09). Recorded patterns included controlled extracranial disease, uncontrolled disease at the primary site only, uncontrolled oligometastatic disease defined as five or fewer extracranial metastases, and uncontrolled widely metastatic disease. Histological distribution varied between groups (p = 0.04), though non-small cell lung cancer represented the majority of cases in both cohorts.

Treatment variables were similar between the 12-week and six-week MRI cohorts. The median number of lesions treated was two for both groups (p = 0.26) (Table 2). The distribution of lesion locations did not differ significantly (p = 0.29), and the cerebrum was the most frequent site of metastatic disease. Radiation given as an adjuvant treatment to surgery was comparable between groups, with 56 (45.5%) of patients in the 12-week cohort and 53 (48.6%) in the six-week cohort receiving therapy in the post-operative setting (p = 0.69). Most patients were not receiving concurrent systemic therapy, defined as any chemotherapy or targeted agent received within seven days before or after treatment (Table 2). All patients were treated in one to five fractions, and those in the 12-week cohort were more frequently treated in three fractions compared to one fraction in the six-week cohort, though this difference was not significant (p = 0.17).

**Table 2:** Treatment variables. Gy, Gray.

Treatment variables	12-week MRI (n = 123)	6-week MRI (n = 109)	p-value
Median number of lesions treated (range)	2 (1-20)	2 (1-14)	0.26
Location of treated lesions (% distribution in cohort)	-	-	0.29
Cerebrum	64	72	-
Cerebellum	29	20	-
Brainstem	6	5	-
Calvarium	1	3	-
Any lesion treated in the post-operative setting (n (%))	56 (45.5)	53 (48.6)	0.69
Patients on concurrent systemic therapy during treatment course (n (%))	33 (27%)	28 (26%)	0.94
Median dose in Gy (range)	24 (16-27)	20 (16-27)	<0.001
Median number of fractions (range)	3 (1-5)	1 (1-5)	0.17

**Figure 2:**
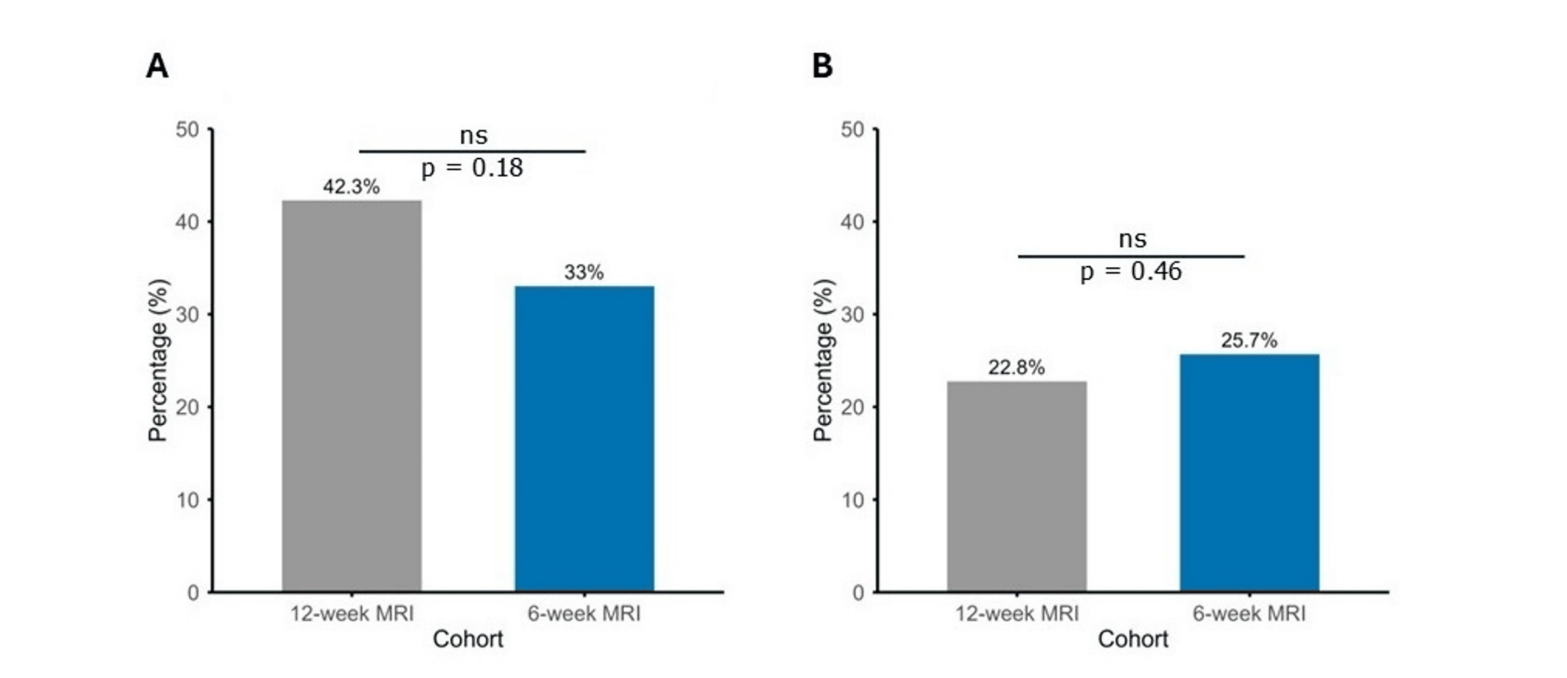
Surveillance MRI findings and presence of neurologic symptoms at the initial follow-up visit. A comparison of the 12-week MRI cohort (gray) to six-week MRI cohort (blue). A) Proportion of patients with MRI findings concerning for progression at the same or distant sites. B) Proportion of patients with new or worsening neurologic symptoms present at initial follow-up. ns: not significant.

Mean time to initial post-treatment surveillance MRI was 91.2 days (SD 24.4) and 43.1 days (SD 7.7) in the 12-week and six-week groups, respectively (p < 0.001). Of patients in the 12-week MRI group, 52 (42.3%) had radiographical findings on MRI concerning for new and/or progressive disease compared to 36 (33.0%) percent in the six-week MRI group (p = 0.18) (Figure 2A). At the corresponding time of clinical follow-up, the presence of new or worsening neurological symptoms was not different between cohorts (28 (22.8%) vs. 28 (25.7%), p = 0.46) (Figure 2B). Neurologic symptoms included new or worsening of pre-SRS/FSR headaches, nausea, vomiting, vision changes including blurry vision or diplopia, focal weakness, focal sensory deficits or paresthesias, imbalance, seizures and altered mental status. Table 3 delineates those with and without evidence of worsening radiographic or clinical findings, including those with the concordant presence of both concern for MRI progression and new or worsening neurologic symptoms. Of patients with MRI progression, 34 (27.6%) in the 12-week cohort and 20 (18.3%) in the six-week cohort had no new or worsening neurologic symptoms (p=0.22).

**Table 3:** Comparison of the number and proportion of patients with and without worsening radiographic and/or clinical findings. Data represented as n (%). Neurologic symptom status was not recorded for four (3%) and seven (6.4%) of patients in the 12-week and six-week cohorts, respectively. Neuro: neurologic.

	Stable or improved	New or worsening
12-week cohort	-	-
MRI findings	71 (57.7%)	52 (42.3%)
Neuro symptoms	91 (74%)	28 (23%)
MRI & Neuro concordance	57 (46%)	18 (14.6%)
6-week cohort	-	-
MRI findings	73 (67%)	36 (33%)
Neuro symptoms	74 (67.9%)	28 (25.7%)
MRI & Neuro concordance	54 (49.5%)	14 (12.8%)

**Figure 3:**
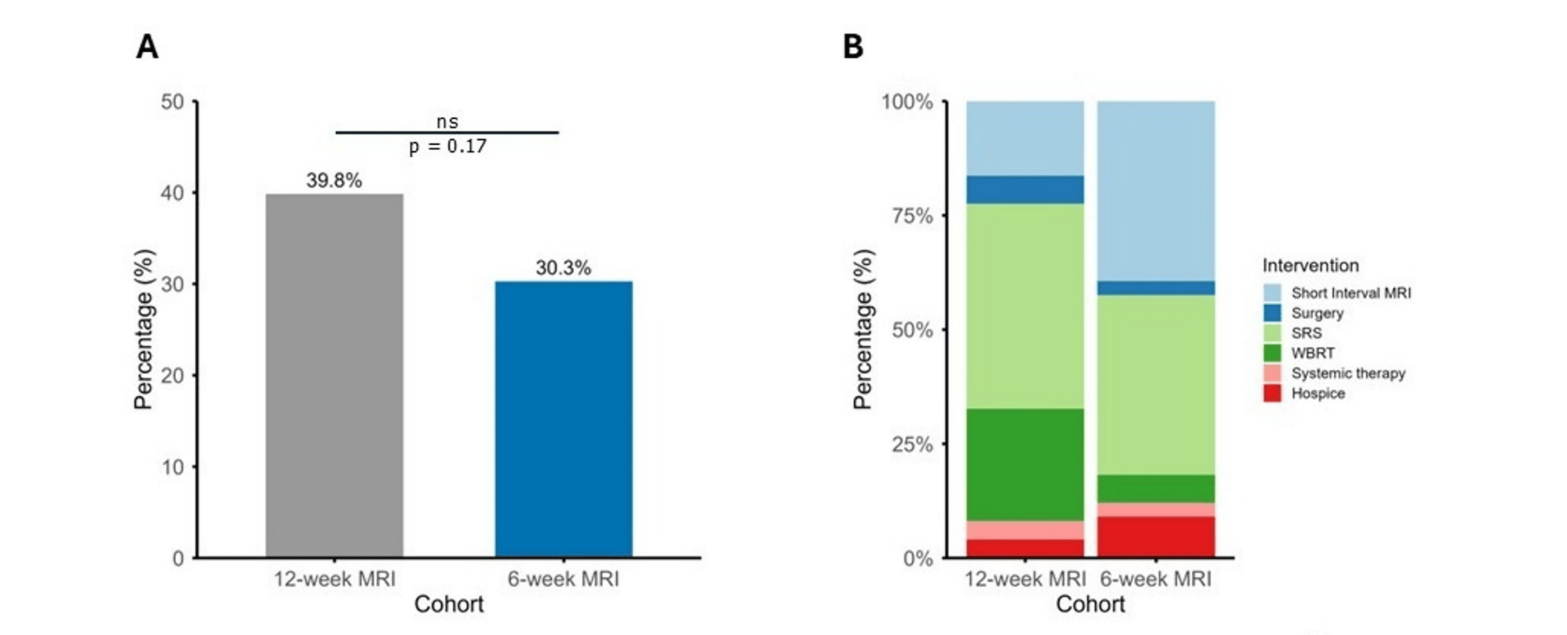
Proportion of patients undergoing intervention following surveillance scans and the types of interventions completed. A) Bar graph showing the proportion of patients in the 12-week MRI cohort (gray) and early six-week MRI cohort (blue) who subsequently underwent an intervention upon completion of follow-up MRI. B) Stacked bar chart showing the type of subsequent intervention completed within each group. SRS, stereotactic radiosurgery; WBRT, whole brain radiation therapy.

Interventions prompted by surveillance MRI findings were recorded. These included repeat short-interval MRI, additional intracranial SRS or FSR, additional intracranial WBRT, surgical resection of new or progressive lesions, addition of new systemic therapy, or enrollment in hospice. Of patients undergoing 12-week surveillance imaging, 49 (39.8%) underwent an intervention which did not significantly differ from the proportion of patients the early imaging group (33 (30.3%)) (p = 0.17) (Figure 3A). In the 49 patients with intervention following surveillance imaging in the 12-week MRI group, 8 (16.3%) underwent a repeat short-interval MRI based on equivocal findings at the time of initial MR, 22 (44.9%) underwent further SRS or FSR, 12 (24.5%) underwent WBRT, 3 (6.1%) underwent surgical resection, 2 (4%) received new systemic therapy, and 2 (4%) entered hospice (Figure 3B). In the 33 patients with intervention following surveillance imaging in the six-week MRI group, corresponding values were 13 (39.4%) short-interval MRI, 13 (39.4%) SRS or FSR, 2 (6.1%) WBRT, 1 (3%) surgery, 1 (3%) new systemic therapy and 3 (9.1%) entered hospice. The distribution of intervention type was not statistically significant between the groups (p = 0.07).

## Discussion

In this study, the radiographic and clinical outcomes following stereotactic brain radiation to brain metastases were compared between two different time points to determine if early surveillance may allow for earlier detection of new brain lesions while asymptomatic and amenable to additional treatment. We show that typical three-month follow-up intervals are clinically appropriate, as no clinically meaningful early detection of progression, either through imaging or symptom-based assessment, was observed in the six-week group.

Most patients treated with SRS or FSR in this cohort (232 of 309) underwent initial recommended surveillance MRI imaging. More patients underwent 12-week surveillance than six-week surveillance, though patient and treatment characteristics were quite similar between groups. Notable differences between the groups include histology type, where the 12-week cohort had more patients with non-small cell lung cancer and the six-week cohort had more patients with melanoma. Additionally, the median prescription dose was 4 Gy higher in the 12-week cohort compared with the six-week cohort, though with the same dose range in both groups; this is likely confounded by different fractionation schemes utilized. Indeed, the biologically effective tumor single-fraction equivalent dose for commonly used fractionation schemes in this study, 24 Gy in 3 fractions and 20 Gy in 1 fraction, is nearly equivalent at 19.7 and 20, respectively [9]. Overall, these group differences are unlikely to have impacted the observed outcomes, which show 12-week surveillance is appropriate.

At initial follow-up, 36 (33%) and 52 (42.3%) of patients in the six- and 12-week cohorts had imaging findings concerning for CNS progression of disease, respectively, either locally or in the untreated brain. A recently published single-center retrospective review evaluated the initial MRI interval post-stereotactic brain radiation, with a median of 57 days to MRI, at which 22.5% of patients were found to have new brain metastases or leptomeningeal disease [10]. Another study evaluating new brain metastasis formation following SRS showed a median time to development of 8.8 months, with 36% of patients having distant brain lesions at six months post-SRS [11]. In randomized controlled trials, intracranial tumor control with SRS alone has been recorded 75% at three months [1], 50-65% at six months [1,4], and 27-50% at one year [1,2], and in the adjuvant setting, 80% at three months [3]. At first glance, the proportion of patients within our study groups with concerning findings on the initial MRI scan may appear high. However, these prospective trials limited patient participants to those with one to four metastases, with size restrictions, without lesions in the brainstem, with a favorable performance status, often with no prior brain radiation treatment, and with restrictions on histology types included. Patients treated in practice often do not conform to randomized trial criteria, creating challenges to study the optimal management of the cranial component of metastatic disease. Our data included nearly one-quarter of patients with a prior history of brain radiation, 12% with poor performance status, and a minority of patients with extracranial disease controlled at the time of treatment. Additionally, our MRI outcome included patients whose findings were equivocal or may represent local pseudoprogression. Indeed, 32% of metastatic brain lesions treated with SRS have been found to increase in size beginning at six weeks post-SRS [12]. Previous data from our institution show excellent post-SRS or FSR local control rates consistent with others [9,13,14].

Most patients with concerning MRI findings underwent subsequent intervention, as expected. The presence of neurological symptoms at the time of clinical follow-up did not statistically differ between the groups. Neurological symptoms included new or worsening headaches, a known side effect of brain radiation treatment, though a recent prospective observational study found that SRS-related headaches were seen in 39.9% of patients, with the majority peaking within two weeks after SRS and resolving within three months [15]. In the present study, a greater proportion of patients in both cohorts had evidence of MRI progression without associated neurological symptoms rather than with clinical findings suggestive of progression, highlighting the importance of surveillance scans. Of the interventions completed, compared to patients with early surveillance, a greater proportion of patients in our study with 12-week MRI underwent additional radiotherapy (SRS, FSR, or whole brain). In a recent retrospective study of patients with brain metastases treated with SRS, 22% were re-treated with SRS within the study period [16]. Further surgical intervention or systemic therapy changes were similar between groups in our study. Within the six-week cohort, a higher proportion of patients went on to complete short-interval MRI compared with the 12-week cohort, which was recommended in the cases of equivocal findings on initial MRI scan. Thus, while early surveillance resulted in more frequent follow-up imaging needs, it did not result in an increase in initial treatment interventions compared to the longer imaging timeline. Based on these results, we conclude that there are no clinically adverse consequences of waiting to detect progression with typical three-month follow-up patterns in many cases.

This retrospective clinical study has inherent limitations. Firstly, although most patients had MRI surveillance imaging available and were eligible participants, one-quarter lacked follow-up imaging, introducing selection bias toward patients who remained well enough for and/or complied with scheduled evaluations. Secondly, differences in tumor biology between the non-randomized groups, such as histology type as discussed above, could confound results. However, because the primary outcome was imaging-based rather than survival-based, systemic factors may be less influential. Another limitation of the study is that the initial post-treatment scans were scheduled approximately six weeks or 12 weeks following completion of radiation therapy based on the practice patterns of different providers, a variable that could impact outcomes aside from timing alone. However, this also reduces selection bias because scan timing reflected provider preference rather than clinical concern for higher risk patients. Indeed, treatment variables were highly similar between the cohorts.

Our study emphasizes the safety of a three-month surveillance imaging interval following SRS or FSR in this cohort of patients with intact or resected brain metastases. In a study assessing the cost-benefit of MRI follow-up in a population of patients with brain metastases termed long-term survivors, there were 373 patients analyzed and only 55, or 15%, of patients who had a year or more of follow-up data available and did not have any CNS failure during the first year post-treatment [7]. This illustrates that, while overall survival for patients with brain metastases is increasing, still many patients progress or succumb to disease within the first year. Identifying patients who are at high-risk for early brain progression may identify a group that could benefit from early surveillance when new lesions are asymptomatic and can be treated. For example, a recent study found that patients with gastrointestinal primary cancers have significantly shorter intracranial progression-free survival than non-gastrointestinal primaries, with most failures occurring in the distant brain [17]. In a study of surveillance MRI timing and survival impact, whether MRI occurred within days 30-60 or 60-90 following radiation did not affect survival; however, patients with early distant brain failure identified on initial scan had worse survival outcomes [10]. A predictive nomogram was developed for patients with brain metastases treated with SRS to determine time to intracranial progression using the number of treated metastases, history of prior WBRT, and time from cancer diagnosis to initial metastases as factors for stratifying patients into low-risk or high-risk groups [18]. Additional studies are warranted to validate predictive models, identify additional high-risk factors and analyze the outcomes in SRS and FSR modalities to better understand the optimal post-treatment imaging surveillance intervals and tailor the interval to reflect individual patient needs.

## Conclusions

Among patients with brain metastases treated with stereotactic radiation, patients undergoing 12-week initial surveillance MRI had a higher incidence of new or progressive disease translating to higher interventions completed compared to patients undergoing six-week MRI, though not statistically significant. As the presence of neurological symptoms was similar between the groups, these data suggest that surveillance MRI at 12 weeks is clinically acceptable and does not increase risk of development of symptomatic metastases relative to six-week surveillance in most cases. Further studies may identify high-risk subgroups that would likely benefit from early surveillance, offering a personalized surveillance strategy for these patients. 
